# Gender- and age-specific associations between psychosocial work conditions and perceived work sustainability in the general working population in Taiwan

**DOI:** 10.1371/journal.pone.0293282

**Published:** 2023-10-25

**Authors:** Yawen Cheng, Yi-Jing Li, Wan‐Ju Cheng

**Affiliations:** 1 Institute of Health Policy and Management, College of Public Health, National Taiwan University, Taipei, Taiwan; 2 National Center for Geriatrics and Welfare Research, National Health Research Institutes, Miaoli, Taiwan; 3 Department of Psychiatry, China Medical University Hospital, Taichung, Taiwan; 4 Department of Public Health, China Medical University, Taichung, Taiwan; National Institute for Occupational Health, Division of the National Health Laboratory Services, SOUTH AFRICA

## Abstract

**Objectives:**

One aspect of work sustainability pertains to workers’ intention to remain in their current job until reaching retirement age. Various adverse working conditions are expected to diminish work sustainability among different social groups. This study aims to examine these associations across gender and age groups.

**Methods:**

The study participants were 19,152 economically-active adults in a national survey conducted in Taiwan. Information concerning psychosocial working conditions were obtained through interviews, using the Job Content Questionnaire. Work sustainability was evaluated by one question that asked whether the participants felt they would be able to do their current job until the age of 60. The association between psychosocial work conditions and work sustainability was examined by logistic regression analysis. We further performed stratified analysis to explore age and gender-specific associations.

**Results:**

We observed that 14.2% and 17.1% of male and female workers reported low work sustainability. Workers in the electronics industries and female workers in the healthcare and education sectors reported low work sustainability. Gender-specific analyses showed that low job control among men and shift work among women were significantly associated with low work sustainability. Age-specific analyses indicated that having poor health, shift work, and long working hours in younger workers, and having low job control in older workers were associated with low work sustainability.

**Conclusion:**

To retain older workers in the labor market, policies should aim at the improvement of psychosocial work conditions, and gender- and age-specific issues should be taken into consideration.

## Introduction

Population aging is a global phenomenon which poses multiple threats to the health and economic sustainability of society, not only due to rising expenditures on healthcare and pensions but also shrinking workforces. The demographic shift of aging started in economically advanced countries, but it is now in developing and recently developed countries that are experiencing the greatest changes within a much shorter time span. Taiwan is among the fastest-aging countries in the world, largely due to extremely low total fertility. The size of the population over 65 surpassed 14% in 2018 and is expected to surpass 20% by 2025. However, labor force participation rates among older people were 62.2% in men aged between 55 and 64 and below 35% among women in the same age range in 2021. To remain economically sustainable and to promote active aging, it is imperative to incorporate work retention elements into labor policies both at organization- and country-levels. Research is needed to identify specific industrial sectors that are less likely to retain middle-aged and older workers. It is also important to consider the influences of psychosocial work conditions on peoples’ work motives, as well as the roles of gender and age for work sustainability.

Cross-sectional studies from European countries have identified a number of factors that were correlated with workers’ intention to early retirement, including low job control, low work-related social support, job insecurity, high workload, and lack of work skills and knowledge [1–5]. Relative to early retirement, the concept of work sustainability concerns more about the motivation to keep working. “Sustainable work” was defined by the European Union (EU) as “achieving living and working conditions that support people in engaging and remaining in work throughout an extended working life” [6]. In the 6^th^ European Working Conditions Survey (EWCS) report, “sustainable work” involves two aspects. One concerns the quality of work that should ensure workers’ capacities and motivation to continue working. The other concerns the physical and psychosocial work conditions and external resources that could support workers to continue working. In the EWCS 2015 survey, work sustainability was assessed by a single question that asked workers under 55 years old whether they felt they could do their current job until they reached the age of 60 [6]. Findings from the survey indicated that ergonomic hazards were the most prominent work factor hampering work sustainability. Results from the EWCS also demonstrated that psychosocial work hazards, including work intensity, shift work and night work, job insecurity, unfair treatment, workplace bullying and workplace violence were major predictors of low work sustainability [6].

### Gender inequality in work sustainability

Some studies demonstrated gender inequalities in working conditions and family caregiving burden, making women more vulnerable to exit from paid employment [7, 8]. In Taiwan, findings from the Manpower Utilization Survey conducted in 2019 indicated that among economically inactive women aged between 25 and 64 years, the main reasons for not working were responsibilities for household work (cited by 46%), followed by family care responsibility for young children or older family members (cited by 19.1%) and lack of economic incentive for working [9]. In contrast, among economically inactive men in the same age range, the main reasons for not working were old age (50.3%), lack of economic incentive for working (19.1%), and poor health (11.9%). Earlier studies in Taiwan revealed that female workers experienced higher levels of job insecurity, lower job control, and increased burnout compared to their male counterparts [10]. Societal expectations for women to shoulder household and family care responsibilities may hinder their career advancement while striving for work-life balance. These challenges are particularly pronounced in Taiwan and other East Asian countries, where long working hours and prioritization of work over work-life balance are prevalent in work cultures.

### Aging and work sustainability

Old age may have direct impacts on work sustainability. Research by Harris et al. indicated that older workers were less likely to be reemployed after being laid off, more likely to become long-term unemployed, and when reemployed, were more likely to enter low-income and low-skilled jobs [11]. Poor health is the major reason for work discontinuation among older workers [12, 13], and maintaining a balance between work and health has been found to improve work sustainability among older workers [14]. While work ability decreases by age, having decision authority, meaningful work content, and personal resources (e.g., hope and resilience) are found to mitigate the negative impacts of aging on work ability [15]. From a life course perspective, work sustainability should be promoted in the early stages of working life, given that young workers may be more susceptible to adverse working conditions during career development, and work-related hazards tend to accumulate into later stages of working life. However, age-specific relationships between psychosocial work conditions and work sustainability have not been examined across different age groups.

In response to the aging workforce, Taiwan and many economically advanced countries have launched aging-friendly policies aiming to promote employment in middle-aged and older populations. Nevertheless, the influences of psychosocial working conditions on workers’ intention to stay at work until old age has not been well-understood, especially in a non-western cultural context. In this study, we assessed workers’ self-reported work sustainability using the same item used in the sixth EWCS, that asked respondents whether they felt they could do their current job until they reached the age of 60. The level of work sustainability was examined across industrial sectors and by psychosocial work conditions. The study aimed to provide empirical evidence for the designs of employment promotion policies that take age and gender-specific aspects into consideration. We hypothesized that adverse work conditions, specifically long working hours, shift work, low job control, high job demands, and job insecurity, were associated with low work sustainability, with variations based on gender and age.

## Materials and methods

### Study participants

The Ministry of Labor of Taiwan has conducted nationwide surveys of the working population every 3–5 years since 1988 to understand safety and health conditions in the workplace. For each survey, participants were selected through a two-stage random sampling process. In the first stage, all districts and villages throughout Taiwan were grouped into strata according to their levels of urbanization. In the first stage, a random sample of districts and villages was selected from each stratum. In the second stage, households were randomly chosen within each district or village, and individuals currently employed at the time of the survey were identified from the sampled households. Subjects who were not economically active were not eligible. The surveys were conducted face-to-face by trained interviewers. More detailed information with regard to the sampling scheme can be found elsewhere [16]. In this study, we utilized data from the survey in 2010. The response rates for this wave of survey were 87% with a total of 23,932 participants. Given the low labor participation rates for individuals older than 55 and the transitional employment stage for those under 25, our study focused exclusively on workers in the age bracket of 25 to 55. We included 19,398 participants (11,209 males and 8,189 females) with an age between 25 and 55 years old. The data were accessed on December 28, 2019 for research purposes. The authors had no access to information that could identify individual participants during or after data collection.

### Ethics statement

This research relied exclusively on secondary use of anonymous information, and the process of data linkage or recording or disseminating of results does not generate identifiable information. This study posed no potential risks to individuals or individual privacy. Therefore, this study is exempt from human ethics review. The authors certify that the study was performed in accordance with the ethical standards as laid down in the 1964 Declaration of Helsinki and its later amendments or comparable ethical standards.

### Measurements

In the questionnaire, participants were asked “Do you feel that you could do your current job until the age of 60?” The respondents chose from “probably could”, “probably could not”, “not sure”, and “unwilling”. We categorized the participants into the low work sustainability group if the responses were “probably could not” or “unwilling”. Other responses (“probably could” and “not sure”) were placed into the high work sustainability group. The response was missing for 246 participants, so the remaining 19,152 workers (11,066 men and 8,086 women) were included in the following analysis.

In regards to work conditions, we measured job control, psychosocial job demands, physical demands, and job insecurity. Job control and psychosocial job demands were assessed using the validated Chinese version of the Job Content Questionnaire (JCQ) based on the job strain model by Karasek and Theorell [17]. The JCQ has undergone validation and reliability testing in the Taiwanese population [18]. Job control refers to the degree of autonomy and decision-making authority an employee has in their work, and psychosocial job demands refer to the aspects of a job that require psychological effort. Due to the space limitation of the questionnaire, items were selected from the JCQ, and the measurement partial items has been proven to be valid [19]. The demands scale included five items (work is fast, work is hectic, excessive workloads, not enough time to get the job done, and must concentrate on the job for a long time). Three items subscale (learning new things, non-repetitive work, making own decisions) was selected from the original 9–item questionnaire of job control. The responses were recorded on a four-point Likert scale ranging from 1 (strongly disagree) to 4 (strongly agree). The mean scores for the 3-item job control subscale and the 5-item demands subscale were then ranked into tertiles (low, medium and high). The cutoff scores for job control were 50.0 and 62.5, while for job demands, they were 46.7 and 53.3.

Job physical demands refer to the physical requirements and exertions associated with a particular job. Job insecurity refers to the perception or fear that one’s employment is at risk or unstable. One item for job insecurity (“my job is secure”) and one for physical demands (my job requires a lot of physical effort) were assessed using a four-point Likert scale. Responses were coded dichotomously as agree (scores 3 and 4) or disagree (scores 1 and 2).

Participants provided information regarding their working hours one week prior to the survey. Long working hours was defined as weekly working hours >48. Participants self-reported their working time arrangement, with a response chosen from fixed work shift, rotating work shift and irregular work shift. Those who chose fixed shift were further asked whether they worked in late evening or nighttime. Shift work was defined as having a work schedule other than fixed daytime work.

The participants’ gender, age, education, and industry type were self-reported. Industry type was further coded according to the 9^th^ Standard Industrial Classification by the Ministry of Labor of Taiwan. Self-rated health (SRH) is a composite indicator for universal dimensions of health and has been found to predict mortality [20]. SRH was assessed by a single-item question “In general, how is your health?” and the answer was recorded on a 5-point scale ranging from 1 (very good) to 5 (very poor). The responses were dichotomized into poor SRH (poor or very poor) and good SRH (moderate, good or very good).

### Statistical methods

We used descriptive analyses to show the distribution of sociodemographic characteristics, working conditions, and SRH. The percentage of low work sustainability in each sociodemographic group was shown. We further examined mean age and the prevalence of low work sustainability in different industry types by gender. Multivariate logistic regression analysis was conducted to investigate the relationship between working conditions, comprising job control, job demands, shift work, working hours, job insecurity, and physical demands, and their association with low work sustainability. The analysis was adjusted for age, sex, education, and SRH. Additionally, it was stratified by age groups and sex to explore potential variations within these subgroups. SAS 9.4 (SAS Institute, Cary, NC, USA) was used for the analyses. The significance level was set at *p* < 0.05.

## Results

Among the 19,152 study participants, 56.6% felt that they could do their current job until the age of 60, whereas 28.0% reported “not sure”, and 15.4% reported low work sustainability. A higher percentage of women reported low work sustainability compared to men (17.1% vs. 14.2%; Table 1), and younger participants more frequently reported low work sustainability compared to older participants. Workers with adverse working conditions, namely low job control, high psychosocial job demands, long working hours, job insecurity, and high physical demands reported low work sustainability more often than workers with good working conditions.

**Table 1:** Demographic and work characteristics of workers (N = 19152) and those with low work sustainability (N = 2946).

	All workers	Low work sustainability	
	N	N	(%)
**Sex**			
Female	8086	1379	(17.1)
Male	11066	1567	(14.2)
**Age (years)**			
25–34	6448	1190	(18.5)
35–44	6148	1035	(16.8)
45–55	6556	721	(11.0)
**Education (years)**			
< = 9	3789	501	(13.2)
9–12	6914	1054	(15.2)
>12	8449	1391	(16.5)
**Job control**			
Low	5679	1059	(18.7)
Medium	9483	1302	(13.7)
High	3948	578	(14.6)
**Job demands**			
Low	7926	948	(12.0)
Medium	5527	738	(13.4)
High	5643	1252	(22.2)
**Shift work**			
Yes	4569	819	(17.9)
No	14525	3750	(14.6)
**Working hours >48**			
Yes	7539	1234	(16.4)
No	11613	1712	(14.7)
**Job insecurity**			
Yes	8787	1609	(18.3)
No	10332	1332	(12.9)
**Physical demands**			
High	10262	1842	(18.0)
Low	8876	1103	(12.4)
**Self-rated health**			
Good	18505	2711	(14.7)
Poor	636	234	(36.8)

Low work sustainability was most prevalent in male workers in the electronic, construction, and public administration and defense industries (Table 2). Among women, low work sustainability was most prevalent in the healthcare, electronic, and education industries. The mean age of workers in the electronic industries were relatively young (34.3 for men and 35.3 for women) among all workers.

**Table 2:** Percentage of workers with low work sustainability in different industries by gender (SD = standard deviation).

	Male (N = 11,066)			Female (N = 8,086)		
		Age	Low work sustainability		Age	Low work sustainability
Industry	N	Mean (SD)	N (%)	N	Mean (SD)	N (%)
Agriculture, forestry, fishing, and animal husbandry	681	45.1 (7.4)	66 (9.7)	202	43.8 (8.0)	21 (10.4)
Manufacturing	2,595	39.7 (8.7)	382 (14.7)	1,391	39.7 (8.7)	202 (14.5)
Electronic	841	34.4 (7.1)	156 (18.6)	565	35.4 (7.6)	154 (21.4)
Construction	1,373	41.5 (8.3)	233 (17.0)	170	39.2 (8.7)	25 (14.7)
Wholesale and retail trade	1,477	40.4 (8.7)	182 (12.3)	1,335	38.4 (8.8)	225 (16.9)
Accommodation and food service	502	39.6 (8.5)	76 (15.1)	676	40.3 (8.9)	114 (16.9)
information and mass communication	240	38.8 (8.7)	23 (9.6)	151	36.2 (8.2)	28 (18.5)
financial and real estate	403	39.1 (8.0)	50 (12.4)	580	38.4 (8.5)	98 (16.9)
Professional, scientific and technical services	282	39.8 (8.8)	24 (8.5)	261	36.1 (8.9)	33 (12.6)
Other service	1622	41.6 (8.7)	226 (13.9)	895	40.5 (8.9)	152 (17.0)
Public administration and defense	462	41.8 (8.1)	72 (15.6)	356	41.7 (8.2)	40 (11.2)
Education	426	40.8 (8.1)	58 (13.6)	888	38.5 (8.4)	185 (20.8)
medical, healthcare and care-giving services	162	41.6 (8.3)	19 (11.7)	462	35.5 (8.0)	102 (22.1)
Total	11,066	40.4 (8.6)	1,567 (14.2)	8,086	38.8 (8.8)	1,379 (17.1)

Logistic regression analysis results showed that, after adjusting for age, education, SRH, and all other working condition variables, shift work was associated with low work sustainability in women (adjusted odds ratio [AOR] = 1.26 and 95% confidence interval [CI] = 1.10 to 1.46) but not in men (AOR = 1.11 and 95% CI = 0.98 to 1.26; Table 3). Whereas low job control was associated with low work sustainability in men (AOR = 1.34 and 95% CI = 1.14 to 1.57) but not in women (AOR = 1.07 and 95% CI = 0.89 to 1.27).

**Table 3:** Adjusted odds ratio and 95% confidence interval (CI) of work conditions for low work sustainability by gender in logistic regression models. Covariates were mutually adjusted, and bold font indicates p <0.05.

	Men (N = 11066)				Women (N = 8086)			
	%	OR	(95% CI)		%	OR	(95% CI)	
Age (years)								
25–34	30.4	**1.72**	**(1.48,**	**1.99)**	38.1	**1.42**	**(1.20,**	**1.68)**
35–44	32.9	**1.63**	**(1.41,**	**1.88)**	31.0	**1.42**	**(1.20,**	**1.67)**
45–55	36.7	Reference			30.9	Reference		
Education								
< = 9	21.7	0.91	(0.77,	1.07)	17.2	**0.66**	**(0.54,**	**0.82)**
9–12	37.0	0.95	(0.84,	1.09)	34.8	**0.84**	**(0.73,**	**0.97)**
>12	41.3	Reference			48.0	Reference		
Job control								
High	22.4	Reference			18.3	Reference		
Medium	50.9	0.98	(0.84,	1.14)	47.9	0.95	(0.80,	1.12)
Low	26.7	**1.34**	**(1.14,**	**1.57)**	33.9	1.07	(0.89,	1.27)
Job demands								
Low	41.3	Reference			41.8	Reference		
Medium	29.4	1.07	(0.92,	1.24)	28.3	1.02	(0.86,	1.20)
High	29.3	**1.57**	**(1.37,**	**1.80)**	29.9	**1.74**	**(1.50,**	**2.01)**
Shift work	25.7	1.11	(0.98,	1.26)	21.5	**1.26**	**(1.10,**	**1.46)**
Working hours >48	43.0	1.11	(0.99,	1.24)	34.4	1.11	(0.97,	1.26)
Job insecurity	44.7	**1.47**	**(1.31,**	**1.65)**	47.7	**1.19**	**(1.05,**	**1.35)**
High physical demands	59.8	**1.43**	**(1.26,**	**1.62)**	45.1	**1.51**	**(1.32,**	**1.72)**
Poor SRH	3.3	**2.99**	**(2.37,**	**3.78)**	3.4	**3.37**	**(2.60,**	**4.37)**

In regards with age, low job control was associated with low work sustainability among workers aged 45–55 years old (AOR = 1.61 and 95% CI = 1.27 to 2.05) but not younger ones (Table 4). Whereas shift work (AOR = 1.41 and 95% CI = 1.22 to 1.63), long working hours (AOR = 1.30 and 95% CI = 1.13 to 1.49), and job insecurity (AOR = 1.41 and 95% CI = 1.23 to 1.62) were associated with low work sustainability only in workers aged 25–34 years old.

**Table 4:** Adjusted odds ratio and 95% confidence interval (CI) of work conditions for low work sustainability by age groups in logistic regression models. Covariates were mutually adjusted, and bold font indicates p <0.05.

	25–34 years old				35–44 years old				45–55 years old			
	%	OR	95% CI		%	OR	95% CI		%	OR	95% CI	
Female sex (reference: male)	47.8	**1.26**	**(1.10,**	**1.44)**	40.8	**1.23**	**(1.07,**	**1.42)**	38.1	**1.30**	**(1.10,**	**1.53)**
Education (years)												
< = 9	7.1	0.83	(0.64,	1.09)	16.2	0.87	(0.70,	1.07)	35.6	**0.72**	**(0.58,**	**0.89)**
9–12	31.7	0.92	(0.80,	1.07)	40.8	0.91	(0.78,	1.07)	36.1	**0.80**	**(0.65,**	**0.98)**
>12	61.2	Reference			43.0	Reference			28.3	Reference		
Job control												
High	18.9	Reference			21.9	Reference			21.3	Reference		
Medium	46.8	0.97	(0.80,	1.16)	49.3	0.91	(0.76,	1.09)	52.6	1.05	(0.85,	1.31)
Low	34.3	1.07	(0.88,	1.30)	28.8	1.08	(0.89,	1.32)	26.1	**1.61**	**(1.27,**	**2.05)**
Job demands												
Low	36.4	Reference			40.1	Reference			47.9	Reference		
Medium	29.6	0.96	(0.80,	1.15)	29.0	1.08	(0.90,	1.31)	28.2	1.10	(0.90,	1.35)
High	34.1	**1.58**	**(1.35,**	**1.86)**	30.9	**1.73**	**(1.46,**	**2.05)**	23.9	**1.53**	**(1.25,**	**1.86)**
Shift work	26.2	**1.41**	**(1.22,**	**1.63)**	22.9	1.01	(0.86,	1.20)	22.7	1.02	(0.84,	1.23)
Working hours >48	39.0	**1.30**	**(1.13,**	**1.49)**	39.7	0.97	(0.84,	1.12)	39.4	1.03	(0.87,	1.21)
Job insecurity	48.8	**1.41**	**(1.23,**	**1.62)**	45.3	**1.40**	**(1.21,**	**1.62)**	43.9	1.18	(1.00,	1.40)
High physical demands	52.4	**1.56**	**(1.35,**	**1.80)**	53.6	**1.51**	**(1.29,**	**1.76)**	54.9	**1.37**	**(1.15,**	**1.64)**
Poor SRH	2.6	**4.24**	**(3.08,**	**5.84)**	3.2	**3.02**	**(2.23,**	**4.08)**	4.1	**2.59**	**(1.93,**	**3.48)**

## Discussions

The primary finding of this study is that among Taiwanese workers aged below 55, only 56.6% felt that they could do their current jobs until the age of 60. Although direct comparisons should be made with caution, the level of work sustainability in Taiwanese workers appeared to be lower than in European workers–74% of men and 72% of women reported being able to do their current job until the age of 60 [6]. Moreover, workers in the electronic industry and female workers in the education and healthcare sectors reported low work sustainability. In addition, we observed distinct associations between work conditions and low work sustainability by gender and across age groups. Gender-specific analyses showed that shift work in women and low job control in men were associated with low work sustainability, and age-specific analyses indicated that low job control was associated with low work sustainability among workers aged 45–55 years old, whereas non-standard working hours was associated with low work sustainability among workers aged 25–34 years old.

As Taiwan is currently facing the fastest rate of population aging [21], ensuring a sustainable workforce has become an urgent task. In many European countries, ensuring sustainable and inclusive working conditions over the life course has been the focus of labor and health policies [22]. Some industries, such as the construction industry, were found to have low work sustainability; therefore, workplace interventions have focused on the improvement of physical working conditions such as heavy manual work. For instance, a study on male workers in the construction industry indicated that physically demanding jobs lead to the highest burden of disability [23].

The observation in our study that workers in the electronic industry and women in the education and healthcare industries reported low work sustainability serve as a warning to the future labor force. Taiwan, recognized as a pivotal site for manufacturing high-tech electronic chips and products, hosts a competitive electronic industry marked by a highly-educated workforce, high wages, and generous employment-related benefits. However, workers in the high-tech electronic industry are also known to face heavy workloads, irregular working hours, and a stressful work environment, leading many to consider leaving their jobs due to health concerns [24–26]. Previous studies indicated that workers in this industry face higher risks for various health issues, including skin problems [27], musculoskeletal symptoms [28], circadian rhythm disruption due to shift work [29], cardiovascular diseases [30], and burnout [26]. In this study, we found that workers in the electronic industry were younger compared to those in other industries. While younger age itself was associated with lower employment stability, our findings suggest that high job demands and non-standard working hours might contribute to low work sustainability among workers of the electronic industry.

Low levels of work sustainability are observed among female workers in the education and healthcare industries, which also deserves attention. Many countries were confronted with workforce shortages and low retention rates of nurses, especially younger nurses [31–33]. The high turnover rate among female healthcare workers was due to high psychological job demands and non-standard working hours [34, 35]. Burnout, poor opportunities for development, work-family conflicts, and higher quantitative work demands were the reason for nurses to exit from employment [31]. A qualitative study showed that one of the reasons that women had lower work sustainability compared to men is high emotional demands [36]. Moreover, teachers of primary and secondary education are also at high risks for high emotional demands and subsequent burnout and turnover intention [37, 38]. Given that healthcare and education workers play essential roles in all societies, their working conditions and well-being deserve attention and protection.

Our observation that workers who reported adverse psychosocial work conditions, namely low job control, high psychosocial job demands, shift work, long working hours, job insecurity, high physical demands, more frequently reported low work sustainability compared to those with better work conditions was consistent with previous findings [6, 39, 40]. More specifically, shift work is associated with disability pension and early retirement [32]. For women at all age ranges, having flexible employment arrangements could help extend their working life [7]. Our findings that shift work was associated with low work sustainability in women may reflect the need to care for children and families. In contrast, the finding that low job control was associated with low work sustainability in men but not in women may reflect gender-specific work orientations. For instance, men are generally more inclined to high autonomy and status control, thus, are more likely to pursue managerial jobs. Previous studies also revealed that lack of recognition and development possibilities were associated with the intention to retire in men but not in women [41].

Our findings suggest that working conditions affect work sustainability differently among young and older workers; therefore, meeting their specific needs may help maintain an age-balanced workforce. On the one hand, low job control was associated with low work sustainability in older workers, suggesting that older workers expect more job autonomy and skill discretion after years of work experience. While ageism could be a barrier to extended working life, aging-friendly policies should be adopted to assist old workers’ needs and encourage workplaces to benefit from older workers’ cumulated working experiences. On the other hand, workplace policies should consider the needs of young workers at the stages of career development and family building. Policies to ensure workers’ work-family balance, such as measures to limit shift work and long working hours, should be the focus for improving work sustainability, especially for working parents [42].

A strength of this research was the large study population of representative working people encompassing a wide range of age groups, making it possible to assess age-specific associations between work conditions and work sustainability in different age groups. Past studies concerning work sustainability have focused on the experiences of middle-aged to older workers [2, 43–45], thus might have problems with healthy worker bias, leading to an overestimation of work sustainability [2].

Nevertheless, this study has some limitations. First, the assessment of work sustainability by one question simplified the complex nature of the interaction between individual and environmental factors in deciding whether to keep working or not. Besides, there is a difference between the intention and the ability to continue working, but this study relied on workers’ subjective perception of work sustainability, which combined the two concepts. Second, this study did not include some important factors that would affect work sustainability. In addition to psychosocial work conditions and self-related health, other factors such as family financial status, family care burden, social and labor policy perceptions, and pension system may exert influences on workers’ perceived work sustainability [45, 46]. Third, this study used data from a cross-sectional survey, so the causality between psychosocial work conditions and work sustainability cannot be ascertained. Furthermore, despite the representativeness of the study population, our results may be context-specific and thus cannot be directly generalizable to other societies with different contexts.

## Conclusions

Sustainable work takes a preventive and proactive approach to workers’ life course to enable longer and good quality of work [6]. Labor and health policies should prioritize the promotion of work sustainability, and should commence at the early stages of workers’ careers. Based on our findings, we recommend more policy interventions in specific sectors that are characterized by low work sustainability, including the electronics, healthcare, and education sectors. Furthermore, we argue that the focus of interventions should be on adverse psychosocial working conditions, including shift work, long working hours, and low job control. Age- and gender-specific elements should be taken into consideration.
